# Comparative Analysis of Surgical Durations and Fees Across Eight Types of Glaucoma Surgery Performed by a Single Surgeon

**DOI:** 10.7759/cureus.51675

**Published:** 2024-01-04

**Authors:** Hiroyuki Wakuda, Shunsuke Nakakura, Tomohiro Shojo

**Affiliations:** 1 Ophthalmology, Saneikai Tsukazaki Hospital, Himeji, JPN

**Keywords:** surgical cost, glaucoma surgery, medical cost, surgical time, glaucoma

## Abstract

The duration of several types of glaucoma surgery and reimbursement amounts per minute of surgery remain unknown. This study compared the surgical duration of glaucoma procedures (ab interno trabeculotomy, PreserFlo, ab externo trabeculotomy, bleb revision, EXPRESS, trabeculectomy, Ahmed, and the Baerveldt implant) and their reimbursement amounts in Japan. We retrospectively analyzed 30 consecutive surgeries of each type of glaucoma surgery. The reimbursement amount per surgical hour was calculated by subtracting the implant cost from the total medical fees. Amounts were converted to dollars based on an exchange rate of 1 USD = 133 JPY. The average surgical time was as follows: ab interno trabeculotomy, 7.8 ± 2.1; PreserFlo, 13.5 ± 4.0; ab externo trabeculotomy, 15.2 ± 4.1; bleb revision, 15.6 ± 2.3; EXPRESS, 16.9 ± 2.7; trabeculectomy, 18.5 ± 3.1; Ahmed, 35.8 ± 8.2; and Baerveldt, 39.2 ± 6.2. The reimbursement amounts after implant deduction were as follows: ab interno trabeculotomy, $1,089; PreserFlo, $1,538; ab externo trabeculotomy, $1,430; bleb revision, $259; EXPRESS, $1,600; trabeculectomy, $1,774; Ahmed, $1,600; and Baerveldt, $1,765. Reimbursement amounts per minute varied, with the highest and lowest for ab interno trabeculotomy and bleb revision at $140 per minute and $17 per minute, respectively. Reimbursement amounts per minute of surgery for eight types of glaucoma surgery vary by up to eightfold.

## Introduction

Surgical reimbursement in Japan is standardized nationwide and significantly influences incentives for medical providers while also impacting the practice of medicine in the country [1]. With the aging global population, the economic burden of glaucoma treatment is becoming a concern [2-4]. Glaucoma treatment includes surgery, eyedrops, and laser procedures [5,6]. Glaucoma treatment is mainly focused on lowering the intraocular pressure. Surgery is considered when maintaining sight in the glaucomatous eye is unlikely through medical or laser treatment [6]. Glaucoma surgery can be classified into those that use implants (PreserFlo, EXPRESS, Ahmed, Baerveldt, etc.) and those that do not (trabeculectomy, ab interno trabeculectomy, and ab externo trabeculotomy) [5,7]. As of 2023, the Japanese reimbursement system covers the cost of glaucoma implants that varies depending on the surgical procedure.

The External Preservation Examination Standard, which is based on actual conditions in multiple Japanese medical facilities, provides preliminary estimates for the expected duration and appropriate reimbursement amounts for each type of surgery [8]. However, disparities in reimbursement amounts between medical specialties exist in both Japan and the United States [1,9-12]. In Japan, surgical reimbursement amounts are calculated based on the duration of surgery, technical difficulty, and the number of assistants required [13]. However, the per-hour reimbursement amount for surgeons in Japan is lower than that in the United States, and the variation between the two countries is remarkable [11]. Additionally, the incentives for medical providers based on actual reimbursement and implant prices remain unclear. Tsukazaki Hospital employs a skilled surgeon who can perform all approved types of glaucoma surgery in Japan, enabling a consistent comparative review of the surgical duration and reimbursement amounts for each type of glaucoma surgery.

The objective of this study is to compare the eight types of glaucoma surgery (ab interno trabeculotomy, ab externo trabeculotomy, trabeculectomy, bleb revision, EXPRESS, PreserFlo, Ahmed, and Baerveldt) performed by a single surgeon at a single institution in terms of surgical duration, real-world implant costs, and reimbursement amounts.

## Materials and methods

This retrospective, observational, investigative study received approval from the Ethics Committee of Tsukazaki Hospital (IRB no. 231006) and was conducted in full compliance with ethical principles, including the Helsinki Declaration. Written informed consent was obtained from all participants.

Participants included patients undergoing any of the following types of glaucoma surgery performed by a single surgeon (SN) at Tsukazaki Hospital: ab interno trabeculotomy, ab externo trabeculotomy, trabeculectomy, bleb revision, EXPRESS, PreserFlo, Ahmed, and Baerveldt. For bleb revision surgery, only procedures that involved limbal incision following trabeculectomy and EXPRESS were included. For the Ahmed and Baerveldt procedures, all variants, including anterior, posterior, and vitreous chambers, were included. Exclusions comprised 1) combined surgeries and 2) bleb revision techniques, except for the needle method or limbal incision. Overall, 30 consecutive cases for each type of surgery were retrospectively reviewed from April 9, 2023. SN conducted all surgical procedures. The surgery duration, defined as the time from when the surgeon initiated the procedure with the speculum in place to its removal, was extracted from electronic medical records. The surgical duration for trabeculectomy, EXPRESS, PreserFlo, and bleb revision included the time for the application of mitomycin C (MMC) 0.04%, which was approximately three minutes in our hospital.

Reimbursement amounts for the eight types of glaucoma surgery as of April 2023 were investigated. For implant costs, both the manufacturer’s list price and the actual delivery price to Tsukazaki Hospital as of April 2023 were obtained. Insurance reimbursement covers some of the costs, including implants and sutures. To determine the reimbursement amount from the provider, implant costs were deducted from the reimbursement amount received from insurance. Viscosurgical devices were not considered in this study.

To calculate the reimbursement amount per minute of surgery, the provider’s reimbursement amount was divided by the surgical duration. Amounts were also converted to dollars based on an exchange rate of 133 yen to the dollar (as of April 2023).

Statistical analysis

To investigate differences in surgical duration among the procedures, statistical analyses were performed. Analysis of variance (ANOVA) was used to analyze the surgical duration of all procedures; if results were significant, Tukey’s post hoc test was used to compare the surgical duration among all procedures. The significance threshold was set at 5% for all tests.

Surgical techniques

Ab Interno Trabeculotomy

An incision (1 mm) was made at two sites from the corneal ear side, and the anterior chamber was filled with a viscoelastic substance. Using a gonioscope to visualize the trabecular meshwork, an incision was made with a Tanito microhook, ranging from 120 to 180 degrees [14]. The viscoelastic material was then washed with a balanced salt solution (BSS). The procedure concluded after confirming the non-leakage of aqueous humor from the wound.

Ab Externo Trabeculotomy

Sutures for corneal traction were placed using 9-0 silk to position the eyeball downward. A 7-mm limbal conjunctival incision was made followed by the application of 2% lidocaine with epinephrine to the sub-Tenon for anesthesia and hemostasis. After creating space under both Tenon’s capsule and the conjunctiva to obtain a broad view of the fornix, hemostasis was ensured. Near the limbus, a 4.5 mm × 3.5 mm rectangular scleral flap (half-thickness) was created. Schlemm’s canal was identified at the flap’s edge, into which a trabeculotome needle was inserted. After ensuring the needle’s placement inside Schlemm’s canal, it was rotated toward the anterior chamber and removed. Using 10-0 nylon (MANI, Utsunomiya, Japan), the scleral flap followed by the conjunctiva was sutured.

Trabeculectomy

Similarly to ab externo trabeculotomy, 9-0 silk was used for corneal traction. After creating a 7-mm limbal conjunctival incision, 2% lidocaine with epinephrine was applied to the sub-Tenon for anesthesia and hemostasis. Once a broad fornix view was achieved by creating space under both Tenon’s capsule and the conjunctiva, hemostasis was ensured. A half-thickness 2.5 mm × 2.5 mm square scleral flap was then created. Subsequently, 0.04% MMC was applied for three minutes using a neurosurgical pad sponge underneath both Tenon’s capsule and the conjunctiva followed by rinsing with 100 mL of saline water. A secondary 2.0 mm × 2.0 mm flap was crafted, and a scleral tunnel was initiated. After forming a window in the anterior chamber using a V-lance and Kelly punch, peripheral iridectomy was performed. The scleral flap was sutured using 10-0 nylon with four to five stitches that were manually adjusted to achieve an intraocular pressure of 10-15 mmHg. After suturing the conjunctiva with 10-0 nylon sutures and verifying the non-leakage of aqueous humor, the procedure was concluded [15].

EXPRESS

Similar to trabeculectomy, MMC was applied followed by conjunctival rinsing with 100 mL of saline water. Using a 25-G needle, an insertion into the anterior chamber was made parallel to the iris, after which the EXPRESS device was placed. Subsequently, the scleral flap was sutured with three to four stitches using 10-0 nylon, and intraocular pressure was manually adjusted to 10-15 mmHg by palpation. After suturing the conjunctiva with 10-0 nylon and verifying the non-leakage of aqueous humor, the procedure was concluded [15].

PreserFlo

Using 7-0 silk sutures, the eyeball was retracted at the cornea. Sub-Tenon anesthesia and hemostasis were achieved by applying 2% lidocaine with epinephrine. Space was created under both Tenon’s capsule and the conjunctiva for broad observation followed by coagulation. Using a neurosurgical pad, 0.04% MMC was applied for three minutes under both Tenon’s capsule and the conjunctiva followed by rinsing with approximately 200 mL of BSS. A 1-mm side port was created in the cornea. Using a dedicated knife, an incision was made 3.5 mm from the limbus, ensuring that the knife tip entered the anterior chamber. PreserFlo was inserted, and BSS was injected from the port to confirm flow from PreserFlo. Tenon’s capsule and the conjunctiva were sutured over PreserFlo using 8-0 Vicryl.

Baerveldt

The conjunctiva was incised followed by the application of 2% lidocaine with epinephrine to the sub-Tenon for anesthesia and hemostasis. Two extraocular muscles were identified. After confirming the flow by injecting BSS into the implant, the tube was stented with 6-0 nylon and ligated with 8-0 Vicryl to block BSS outflow. The plate was placed under the identified extraocular muscles. A 23-G needle was used to puncture the eye 3 mm from the limbus, and the desired position was confirmed. The tube was inserted through the needle hole, its placement in the target position was verified, and the tube was fixed to the sclera with 10-0 nylon. A Sherwood slit was then created. The tube and plate were covered with the preserved sclera and sutured with 10-0 nylon. The conjunctiva was sutured with 8-0 Vicryl.

Ahmed

The conjunctiva was incised, and 2% lidocaine with epinephrine was applied to the sub-Tenon for anesthesia and hemostasis. Two extraocular muscles were identified. BSS was injected into the implant to confirm the flow before placing the plate under the muscles. Using a 23-G needle, a puncture was made 3 mm from the limbus, and the desired position was confirmed. The tube was then inserted through the needle hole, and its placement in the target position was verified. The tube was anchored to the sclera with 10-0 nylon. The tube and plate were covered with the preserved sclera and fixed in place using 10-0 nylon. The conjunctiva was sutured with 8-0 Vicryl.

Bleb Revision

A fornix conjunctival incision was made, and 2% lidocaine with epinephrine was applied to the sub-Tenon for anesthesia and hemostasis. The scleral flap was then identified. MMC was applied for three minutes and rinsed with 100 mL of saline water. A 1-mm side port was created in the cornea. The scleral flap was incised at its edge to verify aqueous humor flow. Intraocular pressure was evaluated, and the scleral flap was sutured with as few stitches as possible. The conjunctiva was closed with a continuous suture using 10-0 nylon. Non-leakage of aqueous humor was then confirmed.

## Results

**Figure 1:**
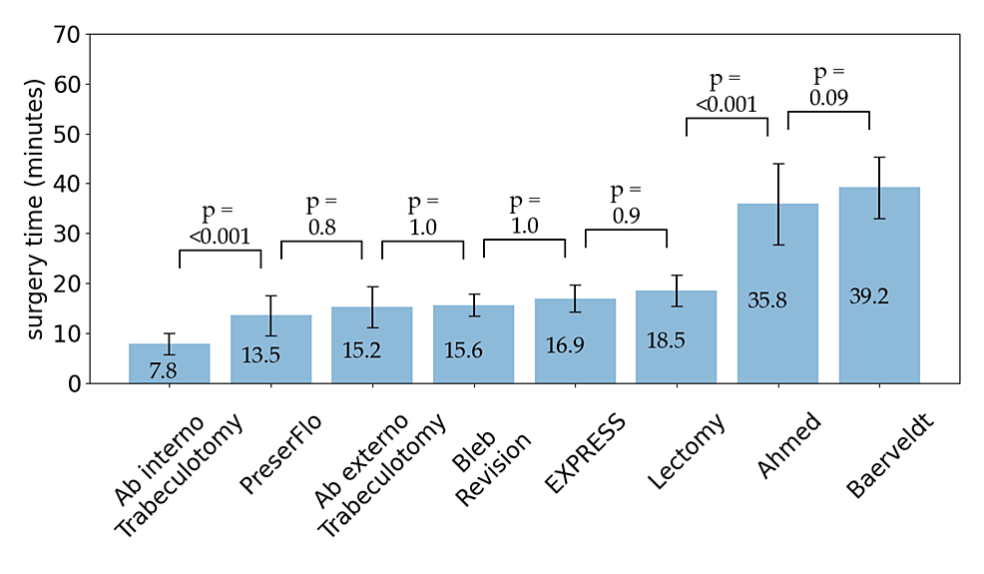
Mean operating time for each glaucoma surgery.

The time to reach 30 cases varied, with trabeculectomy and PreserFlo taking two months, while Ahmed, Baerveldt, and ab externo trabeculotomy took over three years. Background data are listed in Table 1. The average surgical durations (minutes) ± standard deviation were as follows: ab interno trabeculotomy, 7.8 ± 2.1; ab externo trabeculotomy, 15.2 ± 4.1; PreserFlo, 13.5 ± 4.0; EXPRESS, 16.9 ± 2.7; trabeculectomy, 18.5 ± 3.1; bleb revision, 15.6 ± 2.3; Ahmed, 35.8 ± 8.2; and Baerveldt, 39.2 ± 6.2 (Figure 1).

**Table 1:** Characteristics of the study eyes.

		Ab interno trabeculotomy	PreserFlo	Ab externo trabeculotomy	Bleb revision	EXPRESS	Trabeculectomy	Ahmed	Baerveldt
Number of eyes, *n*		30	30	30	30	30	30	30	30
Age, y ± SD		54.1 ± 13.6	71.6 ± 15.8	73.3 ± 14.9	71.2 ± 18.2	78.9 ± 7.5	69.6 ± 11.8	61.8 ± 23.5	71.4 ± 11.9
Male, *n* (%)		20 (67)	18 (60)	11 (37)	14 (47)	14 (47)	16 (53)	14 (47)	20 (67)
Right, *n* (%)		14 (47)	15 (50)	15 (50)	16 (53)	17 (57)	13 (43)	16 (53)	18 (60)
Latest surgery date		April 30, 2022	April 8, 2023	November 7, 2022	April 8, 2023	April 3, 2023	April 9, 2023	April 17, 2022	April 9, 2023
Earliest surgery date		July 21, 2019	February 11, 2023	July 10, 2021	November 3, 2022	April 3, 2022	February 20, 2023	May 26, 2019	December 21, 2020

ANOVA revealed significant differences in surgical duration among all procedures (*F* = 180, *P* < 0.0001). According to Tukey’s post hoc test, the surgical time was significantly shorter for ab interno trabeculotomy (*P* < 0.001) than for any other method, while Ahmed and Baerveldt procedures were significantly longer (*P* < 0.001) than other methods. Moreover, the surgical time for PreserFlo was significantly shorter than that for trabeculectomy (*P* < 0.001); these were the only significant combinations observed.

Reimbursement amounts for glaucoma surgery and the cost of implants as of 2023 are detailed in Table 2. The acquisition cost of implants was lower than the listed price. By subtracting the implant cost from the medical fees, the healthcare provider’s compensation was calculated. After accounting for the cost of the implant, the reimbursement amount per surgery hour varied by approximately eightfold between the lowest price, which was bleb revision, and the highest price, which was ab interno trabeculotomy.

**Table 2:** Cost of each glaucoma surgery in Japan.

		Ab interno trabeculotomy	PreserFlo	Ab externo trabeculotomy	Bleb revision	EXPRESS	Trabeculectomy	Ahmed	Baerveldt
Fee include implant, $		1,089	2,592	1,430	259	2,592	1,774	3,420	3,420
Implant list price, $		-	1,055	-	-	992	-	1,820	1,654
Implant real price, $		-	986	-	-	819	-	1,505	1,075
Fee w/o implant (list price), $		1,089	1,538	1,430	259	1,600	1,774	1,600	1,765
Fee w/o implant (real price), $		1,089	1,606	1,430	259	1,773	1,774	1,915	2,345
Fee per surgery time (list price), $/minute		139.7	113.8	94.0	16.5	94.5	95.9	44.6	45.1
Fee per surgery time (real price), $/minute		139.7	118.8	94.0	16.5	104.8	95.9	53.4	59.8

## Discussion

We demonstrated a discrepancy that was as high as eightfold in the hourly reimbursement amount for glaucoma surgery performed in a single institution by a single surgeon. A substantial disparity exists in medical fees for glaucoma surgery, indicating varied incentives for different procedures. This suggests the potential for an inherent inequality in incentives across Japan when choosing surgical methods to treat glaucoma.

The total and hourly reimbursement amounts for bleb revision were notably lower than other types of procedures. This is despite the surgical duration for bleb revision being comparable to that of trabeculectomy. Due to the markedly low reimbursement amount for bleb revision, inequity may be felt by some surgeons performing this procedure.

Estimates from the external insurance association are derived from field investigations involving multiple medical practitioners [8]. The surgical duration for each glaucoma surgery is reported as follows: ab interno trabeculotomy, 0.5 hour; ab externo trabeculotomy, 1.0 hour; trabeculectomy, 1.0 hour; bleb revision, 1.5 hours; PreserFlo and EXPRESS, 1.0 hour; Ahmed and Baerveldt, 1.5 hours [8]. Comparing these estimates with the actual surgical duration in our study, ab interno trabeculotomy as well as the Ahmed and Baerveldt procedures had a shorter real-time duration. However, the time for bleb revision was longer than the external insurance association’s estimate. Additionally, in Japan, medical fees (including implant fees) were similar for PreserFlo and EXPRESS as well as for Ahmed and Baerveldt; however, there was no significant difference in surgical time among these combinations. In Japan, surgeries with a short duration tend to have a higher reimbursement rate per hour [10], a trend that was also observed in this study.

This study has several limitations. First, it focused on a single surgeon in a single private institution. Hence, the results may not be representative of other hospitals. Additionally, costs per hour might differ based on the facility depending on whether there is an accompanying doctor as an assistant or other medical staff during surgery. The surgical duration may also vary based on the surgeon’s experience and the proficiency of the assistant [16,17]. Regarding the standardization of surgical time for each procedure for the per-minute reimbursement. The surgical time can vary significantly among surgeons and may not always be inversely proportional to the experience of the surgeon. However, this is the first report to show the variety of reimbursement amounts per minute in Japan. Additionally, our results will shed light on the big issue in the growing budget for healthcare in our aged society. Moreover, implant prices might also differ by institution. While this single-institution, single-surgeon study allows for consistent staff support conditions, it may not be representative. Second, merely evaluating the hourly reimbursement amount might not adequately indicate the proper incentives. Apart from the surgical duration, each procedure incurs fixed costs [18], which remain unchanged even for shorter surgeries, suggesting that our metric might be overly simplistic. In Japan, there is a tendency for surgeries with a shorter duration to have a higher reimbursement amount per hour [10]. Moreover, postoperative management requirements differ vastly among procedures, especially for trabeculectomy. While it was difficult to accurately calculate the costs per patient and postoperative management costs, this study did not include these factors. Regardless, the study’s metric of reimbursement amount per surgery hour remains useful due to its simplicity and ease of comparison. Third, some costs, such as sutures and medications, were included in the medical fees but were not considered in this study. Ideally, when calculating the provider’s reimbursement amount, other costs associated with surgery should also be deducted. However, given that implant fees are overwhelmingly higher, they were not considered in this analysis. Fourth, this study did not compare the cost-effectiveness of the surgery from the patient’s perspective. Although previous studies have examined the cost-effectiveness of eyedrops and surgeries [19,20], this study strictly focused on the difference in provider incentives and not the cost-effectiveness for patients. Fifth, the patient characteristics that determine the suitable type of surgery vary for each procedure; some procedures might not be suitable for some patients. Finally, calculations were based on implant prices and medical fees as of April 2023; future changes could lead to different results. In the United States, while there are disparities in reimbursement amounts across specialties, it is assumed that reimbursement amounts are considered based on reports from a few physicians [9]. This study also based its findings on a single physician’s reports. Thus, relying on this study for reimbursement adjustments might introduce inequalities.

## Conclusions

This study provided a comprehensive comparison of the surgical duration and associated reimbursement for eight different glaucoma surgeries performed by a single surgeon in Japan. Findings indicate that, as of 2023, the reimbursement rates per minute of surgery can vary up to eightfold, highlighting significant disparities in financial compensation. Limitations of this study include its focus on a single surgeon's experience, which may not be generalizable to all surgical practices. Further research is needed to confirm these findings across multiple surgeons and institutions.
